# Supplementary material for: NMR, LC-MS Characterization of Rydingia michauxii Extracts, Identification of Natural Products Acting as Modulators of LDLR and PCSK9
Source: Molecules. 2022 Mar 30;27(7):2256. doi: 10.3390/molecules27072256 (PMC9000307; doi:10.3390/molecules27072256)
Supplement: Supplementary file 1 [file molecules-27-02256-s001.zip › molecules-1615727-supplementary.pdf]

NMR, LC-MS characterization of *Rydingia michauxii* extracts, identification of natural products acting as modulators of LDLR and PCSK9.

Supplementary material:

Scheme S1: Mass spectra of compound 1 in negative ion mode (blue spectrum) and MS2 spectra (green spectrum. Proposed structures of main observed ions, indicated by letters are reported.

Scheme S2. tentatively fragmentation pathway in negative ion mode of compound 3

Table S1: NMR assignment for ent-labda-8(17),13-dien-18-oic acid-15,16-olide in deuterated chloroform

Figure S1: ^{1}H NMR of ent-labda-8(17),13-dien-18-oic acid-15,16-olide

Figure S2: HSQC of ent-labda-8(17),13-dien-18-oic acid-15,16-olide

Figure S3: HMBC of ent-labda-8(17),13-dien-18-oic acid-15,16-olide

Figure S4: COSY of ent-labda-8(17),13-dien-18-oic acid-15,16-olide

Figure S5: NOESY of ent-labda-8(17),13-dien-18-oic acid-15,16-olide

Figure S6: TOCSY of ent-labda-8(17),13-dien-18-oic acid-15,16-olide

Table S2: NMR assignment for ent-labda-8(17),13-dien-18-oic acid-15,16-glucopyranoside in deuterated methanol

Figure S7: ^{1}H NMR of ent-labda-8(17),13-dien-18-oic acid-15,16-glucopyranoside

Figure S8: HSQC of ent-labda-8(17),13-dien-18-oic acid-15,16-glucopyranoside

Figure S9: HMBC of ent-labda-8(17),13-dien-18-oic acid-15,16-glucopyranoside

Figure S10: COSY of ent-labda-8(17),13-dien-18-oic acid-15,16-glucopyranoside

Figure S11: NOESY of ent-labda-8(17),13-dien-18-oic acid-15,16-glucopyranoside

Figure S12: TOCSY of ent-labda-8(17),13-dien-18-oic acid-15,16-glucopyranoside

Table S3: NMR assignment for antirrhinoside in deuterated methanol

Figure S13: ^{1}H NMR of antirrhinoside

Figure S14: HSQC of antirrhinoside

Figure S15: HMBC of antirrhinoside

Figure S16: COSY of antirrhinoside

Figure S17: NOESY of antirrhinoside

Figure S18: TOCSY of antirrhinoside

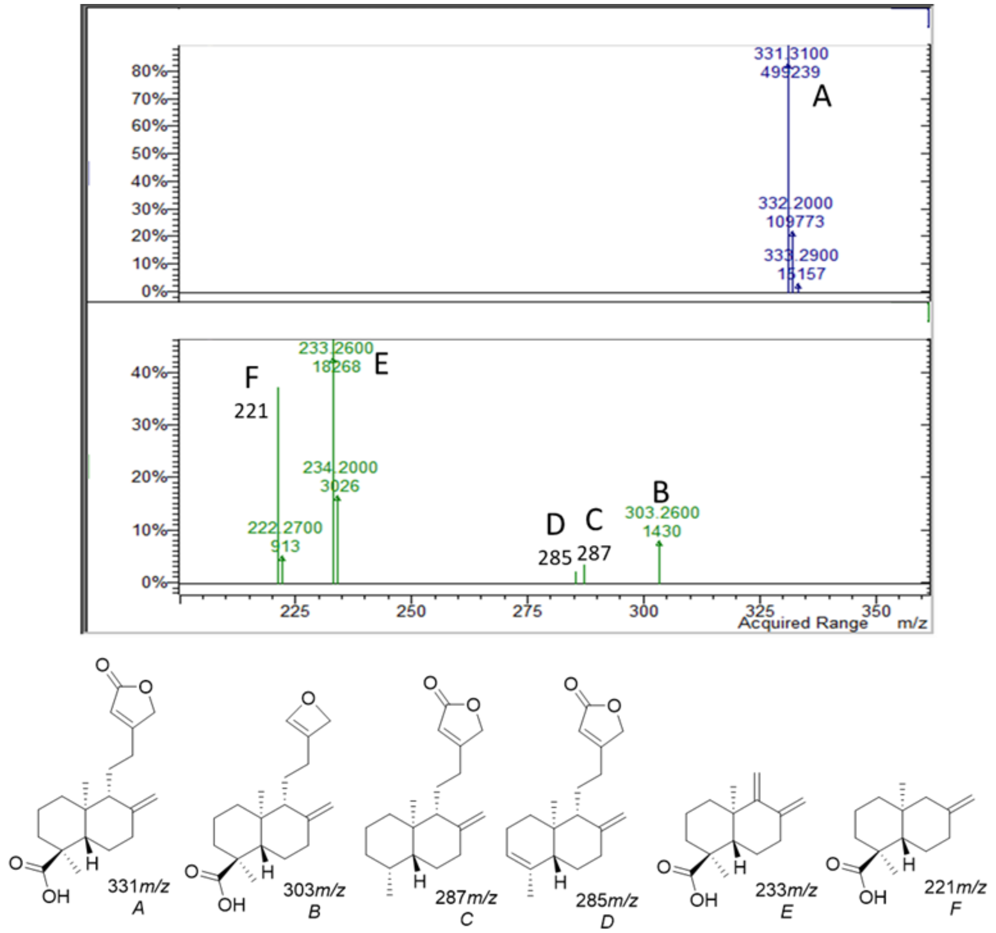

Scheme S1. Mass spectra of compound 1 in negative ion mode (blue spectrum) and MS2 spectra (green spectrum. Proposed structures of main observed ions, indicated by letters are reported.

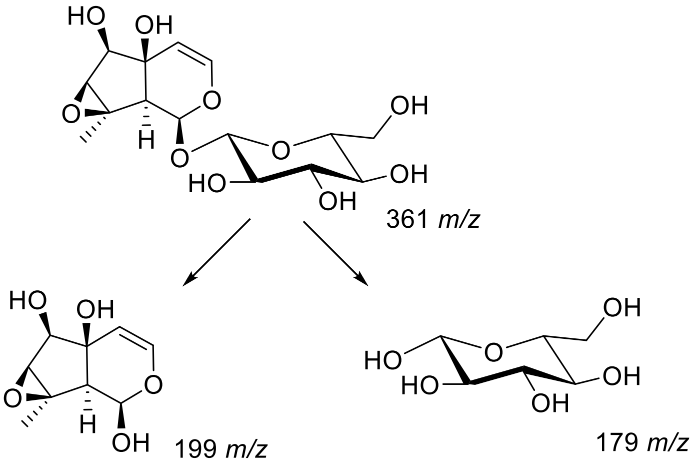

Scheme S2. tentatively fragmentation pathway in negative ion mode of compound 3

Table S1: NMR assignment for ent-labda-8(17),13-dien-18-oic acid-15,16-olide in deuterated chloroform

Position	δH	δC
1	1.17-1.78 m	38.1
2	1.66 m	18.5
3	1.81-1.63	35.5
4	-	47.1
5	1.97m	49.6
6	1.44 m	26.5
7	2.40-2.07m	37.6
8	-	146.2
9	1.74m	56.0
10	-	38.0
11	1.80-1.60m	21.8
12	2.57-2.27 m	27.3
13	-	171.0
14	5.88 s	114.0
15	-	174.6
16	4.76 4.72 d, *J*=18	73.9
17	4.48 *J*=1.0 β<br>4.92 *J*=1.0 *α*	105.8
18	-	185.0
19	1.17 s	16.4
20	0.75s	14.8

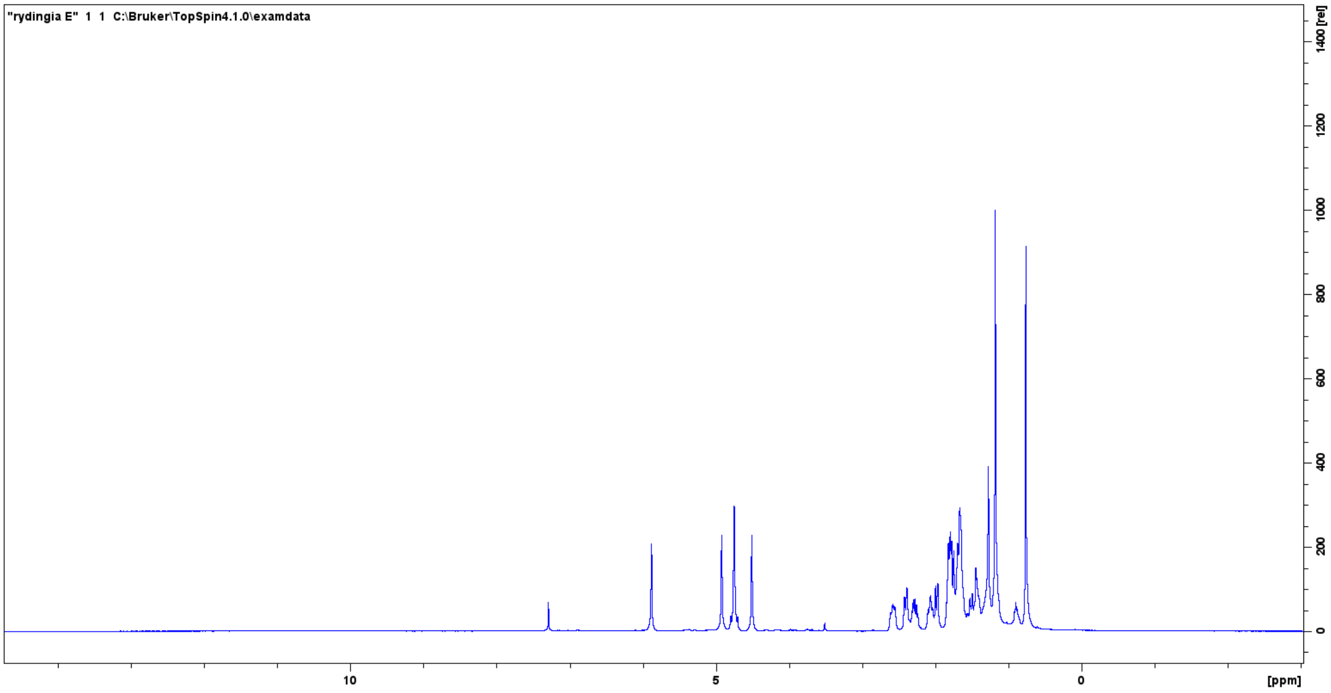

Figure S1: ^{1}H NMR of ent-labda-8(17),13-dien-18-oic acid-15,16-olide

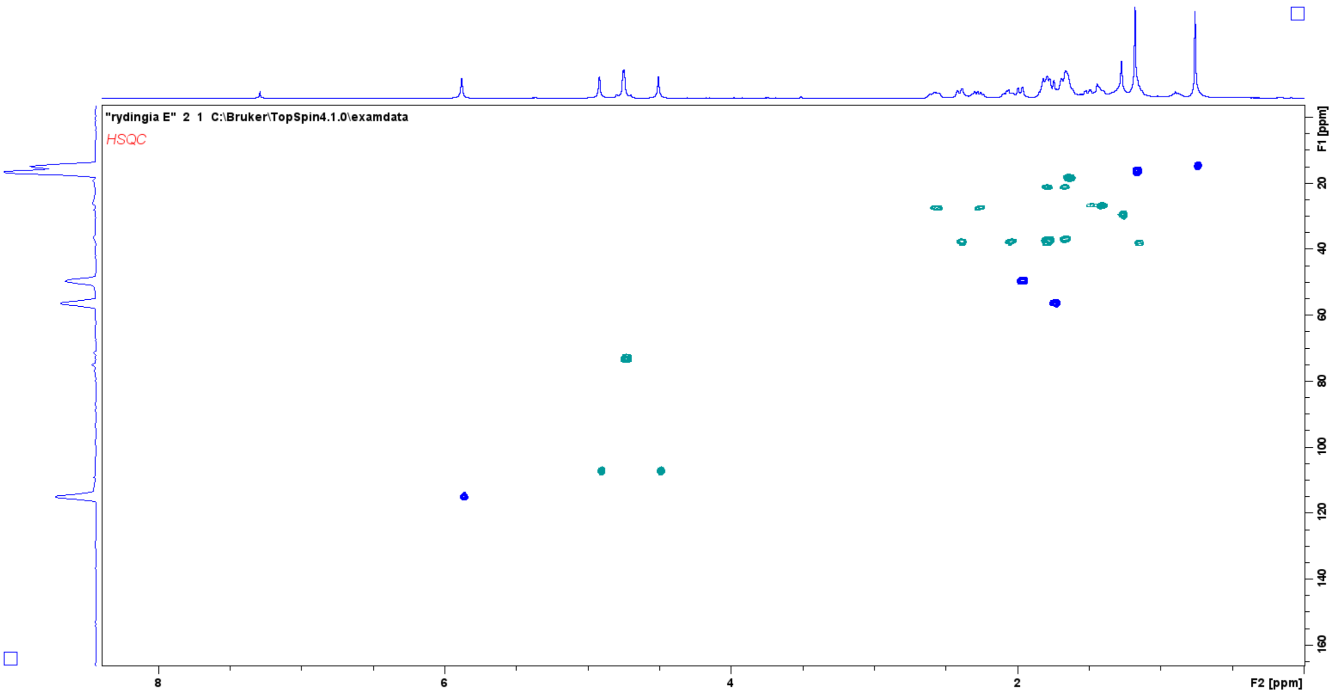

Figure S2: HSQC of ent-labda-8(17),13-dien-18-oic acid-15,16-olide

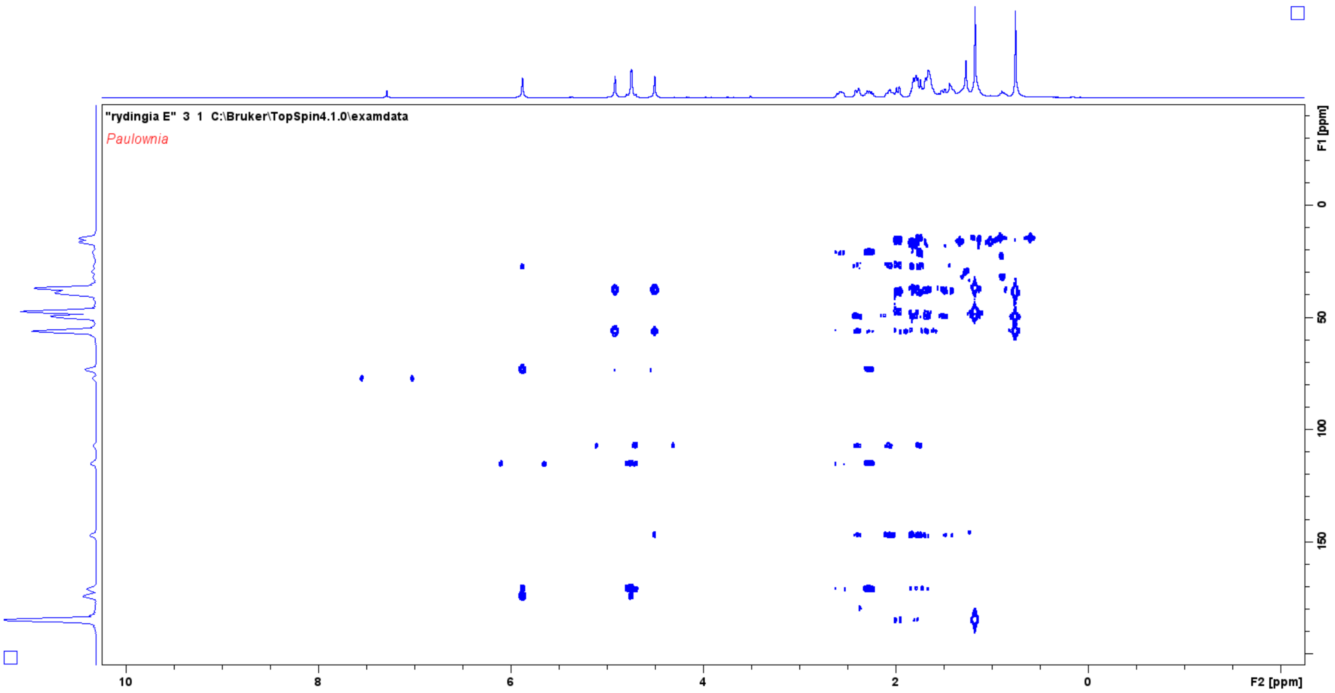

Figure S3: HMBC of ent-labda-8(17),13-dien-18-oic acid-15,16-olide

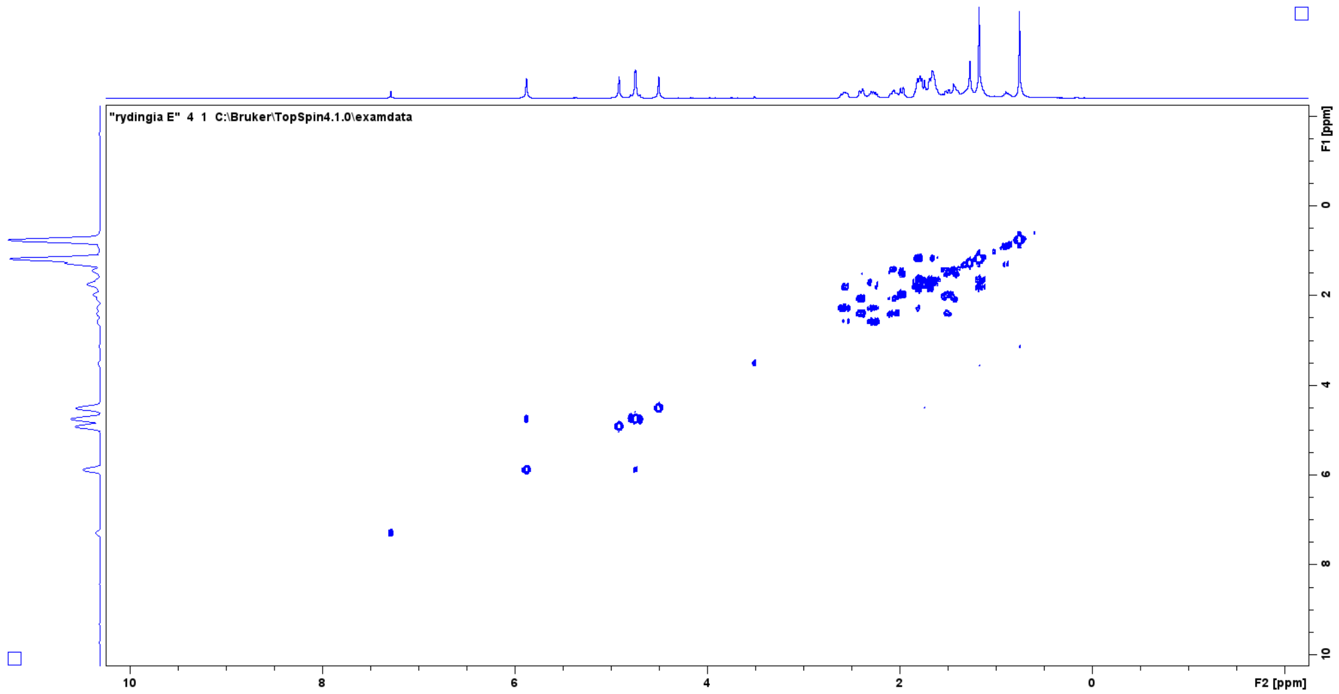

Figure S4: COSY of ent-labda-8(17),13-dien-18-oic acid-15,16-olide

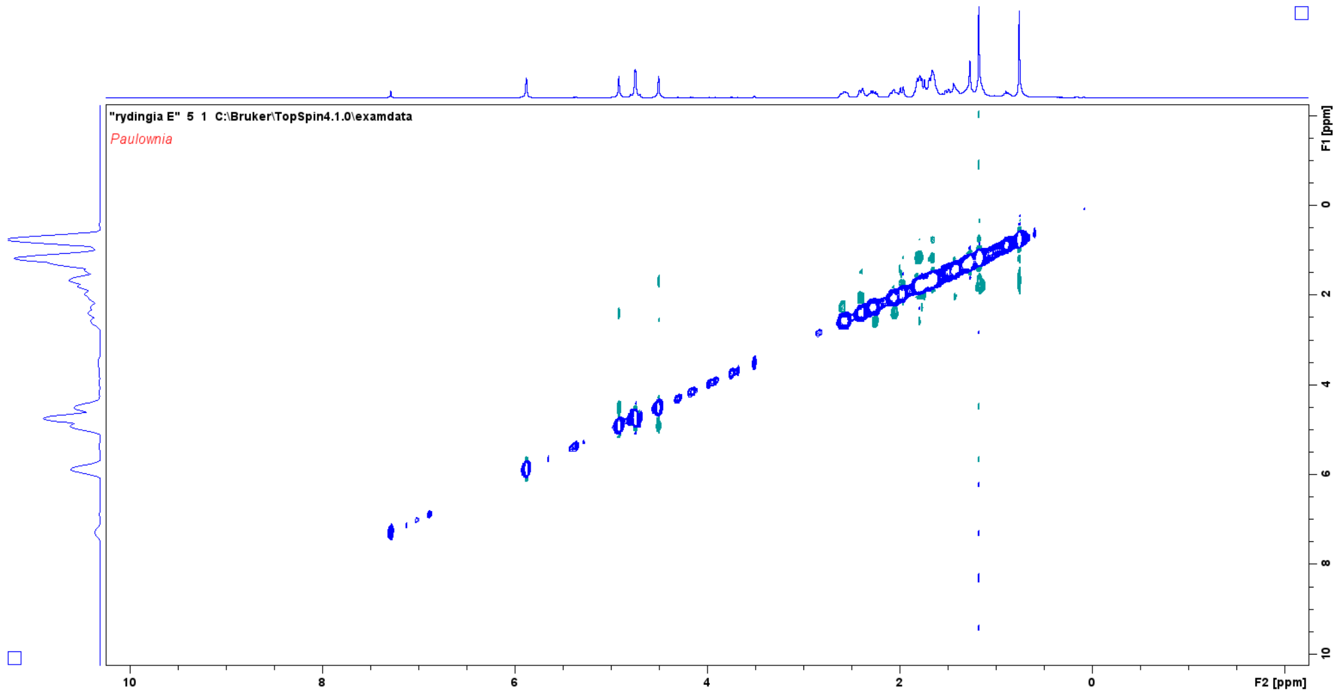

Figure S5: NOESY of ent-labda-8(17),13-dien-18-oic acid-15,16-olide

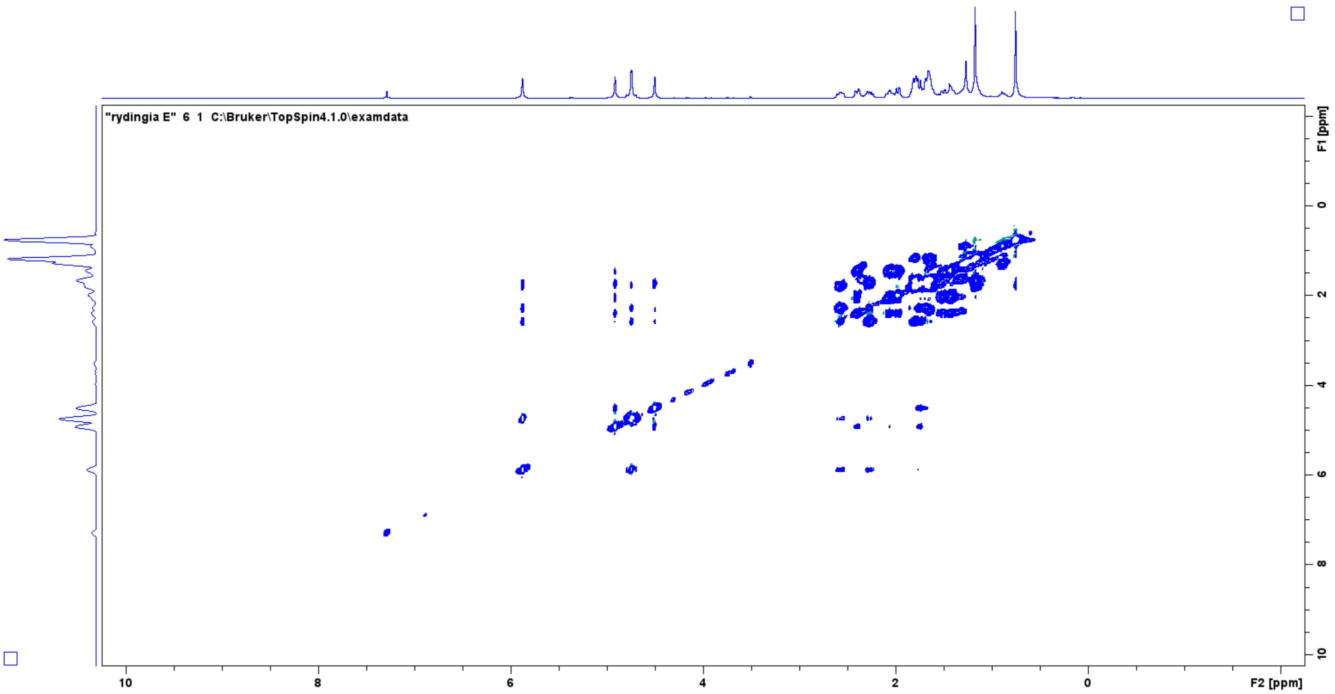

Figure S6: TOCSY of ent-labda-8(17),13-dien-18-oic acid-15,16-olide

Table S2: NMR assignment for ent-labda-8(17),13-dien-18-oic acid-15,16- glucopyranoside in deuterated methanol

Position	δH	δC
1	1.89-1.21 m	37.7
2	1.66 m	18.1
3	1.85 1.63	36.2
4	-	47.4
5	2.10 m	49.3
6	1.42m	26.4
7	2.37-2.12m	37.2
8	-	146.0
9	1.83m	56.5
10	-	38.0
11	1.85-1.74m	21.1
12	2.63-2.33 m	27.4
13	-	171.1
14	5.91 s	114.0
15	-	174.1
16	4.88 under methanol	73.5
17	4.92 *J*=1.0 β<br>4.58 *J*=1.0 α	106.4
18	-	177.4
19	1.20 s	15.7

20	0.79s	14.4
1′	5.45 d, *J*= 8.0	94.8
2′	3.36	72.8
3′	3.46	77.7
4′	3.41	69.9
5′	3.40	77.0
6′	3.87-3.73	61.0

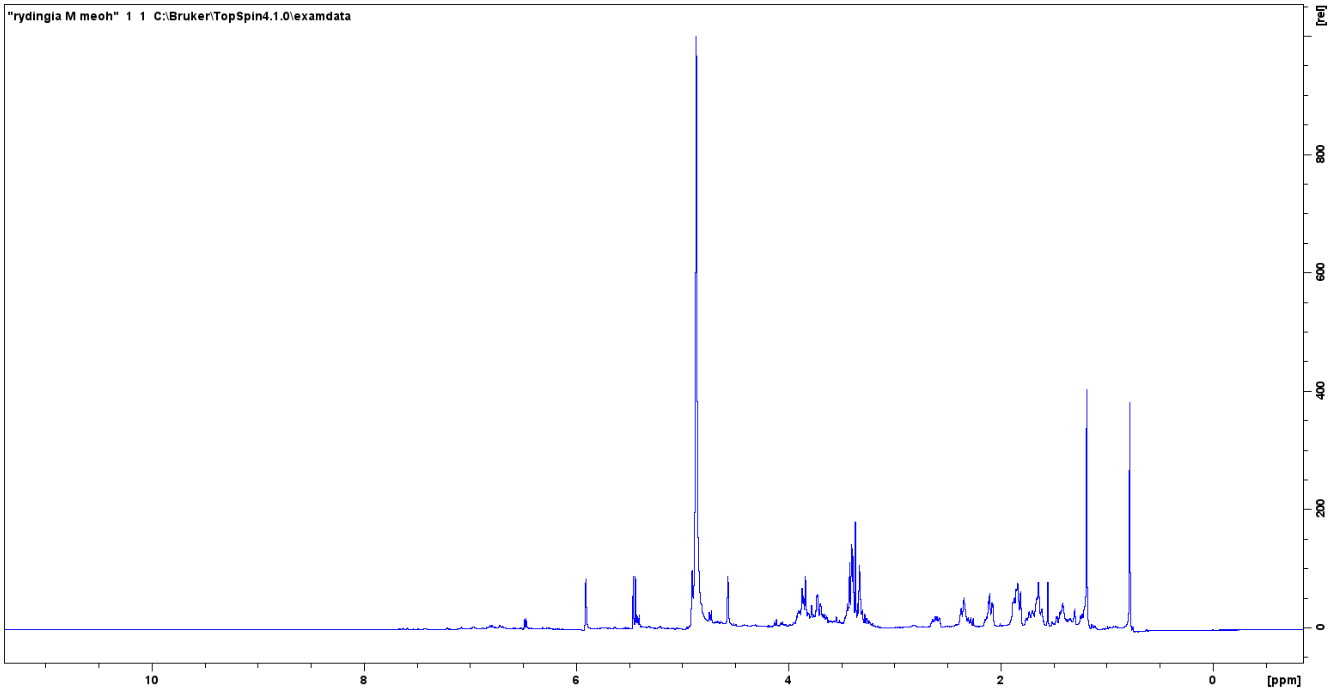

Figure S7: ^{1}H NMR of ent-labda-8(17),13-dien-18-oic acid-15,16- glucopyranoside

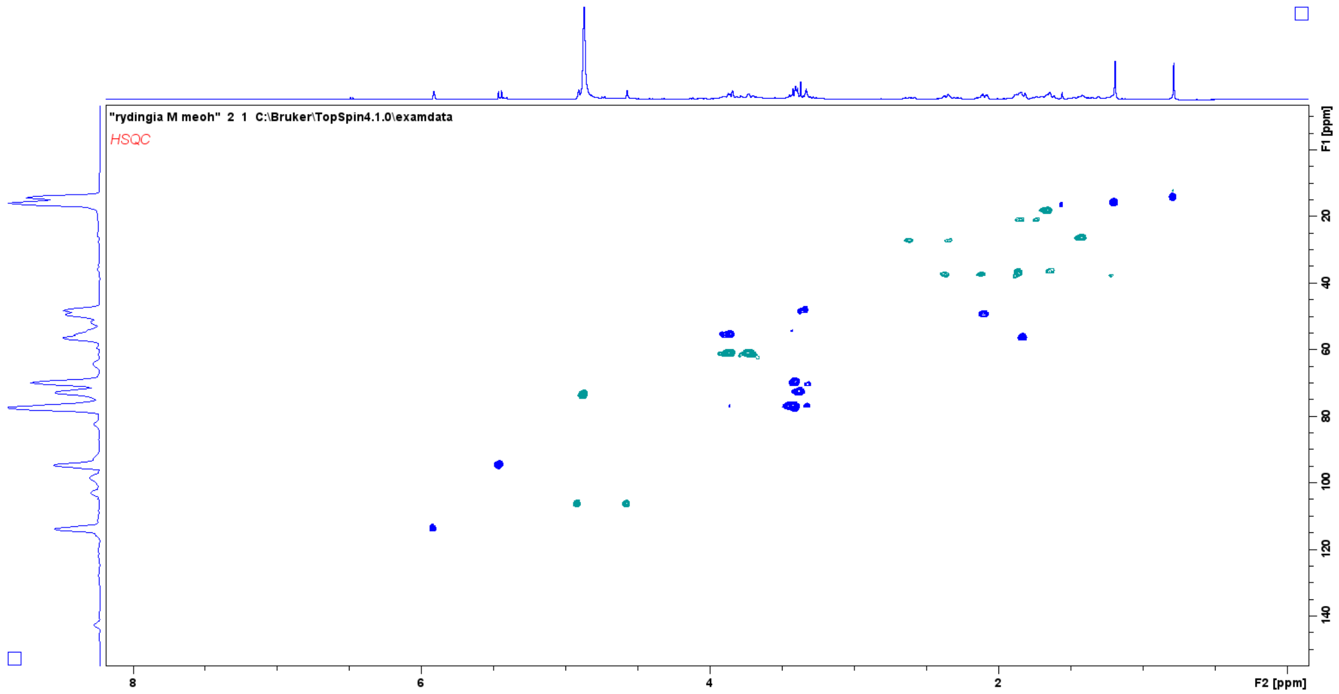

Figure S8: HSQC of ent-labda-8(17),13-dien-18-oic acid-15,16-glucopyranoside

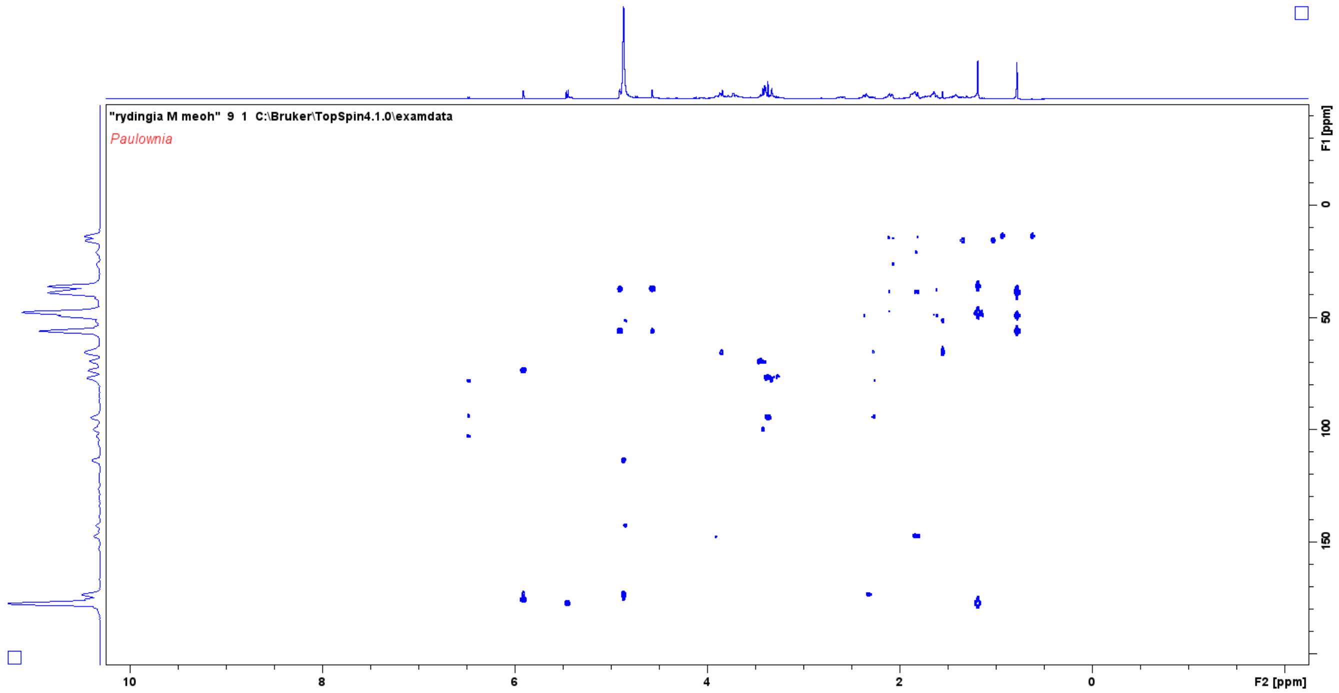

Figure S9: HMBC of ent-labda-8(17),13-dien-18-oic acid-15,16-glucopyranoside

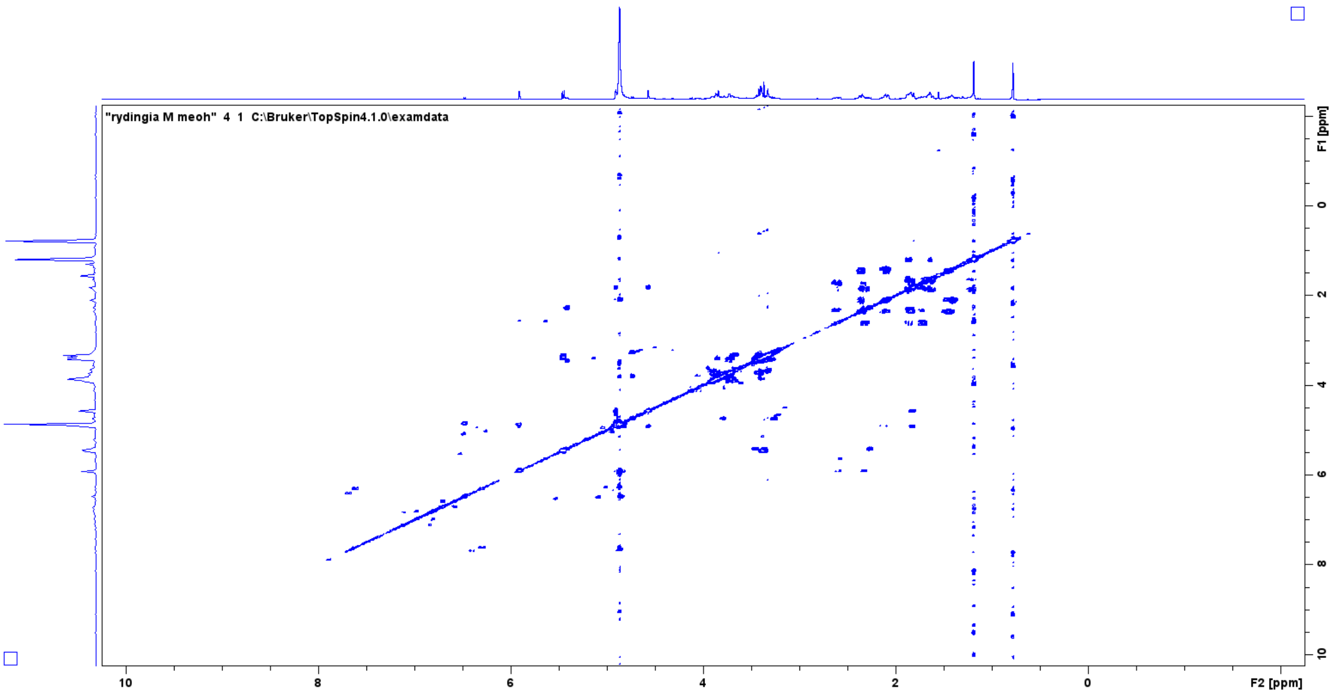

Figure S10: COSY of ent-labda-8(17),13-dien-18-oic acid-15,16-glucopyranoside

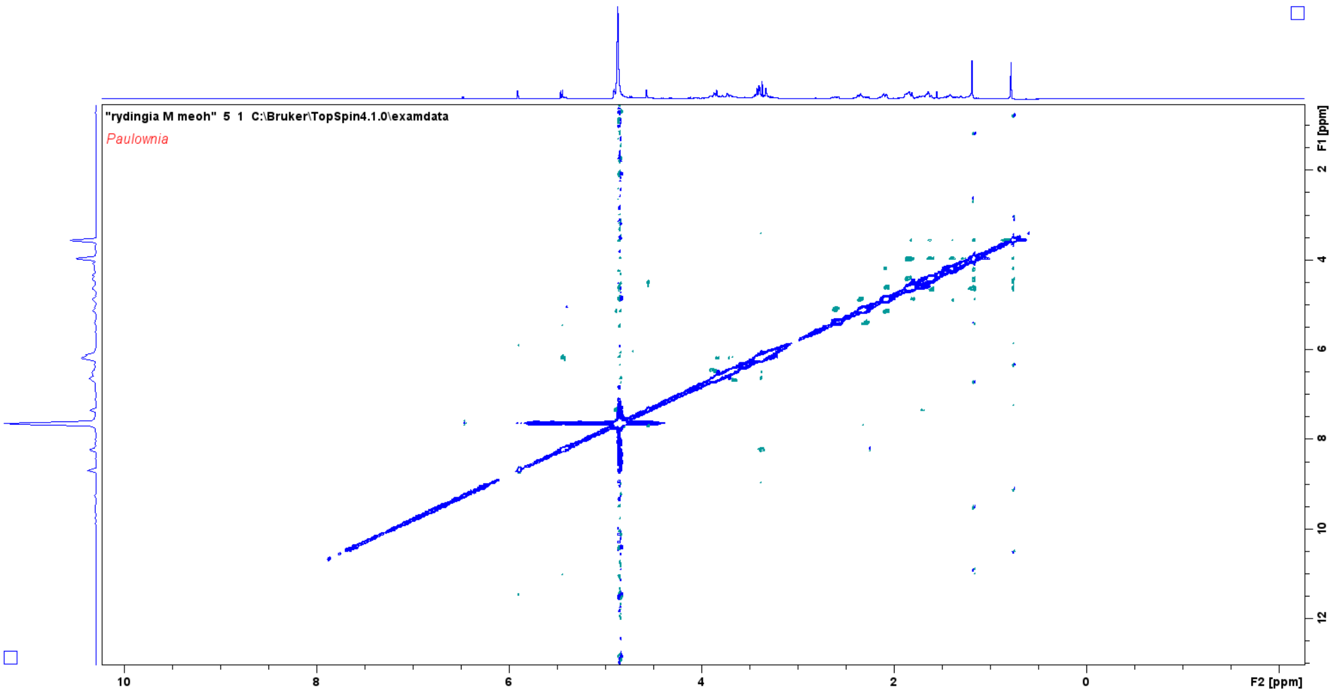

Figure S11: NOESY of ent-labda-8(17),13-dien-18-oic acid-15,16-glucopyranoside

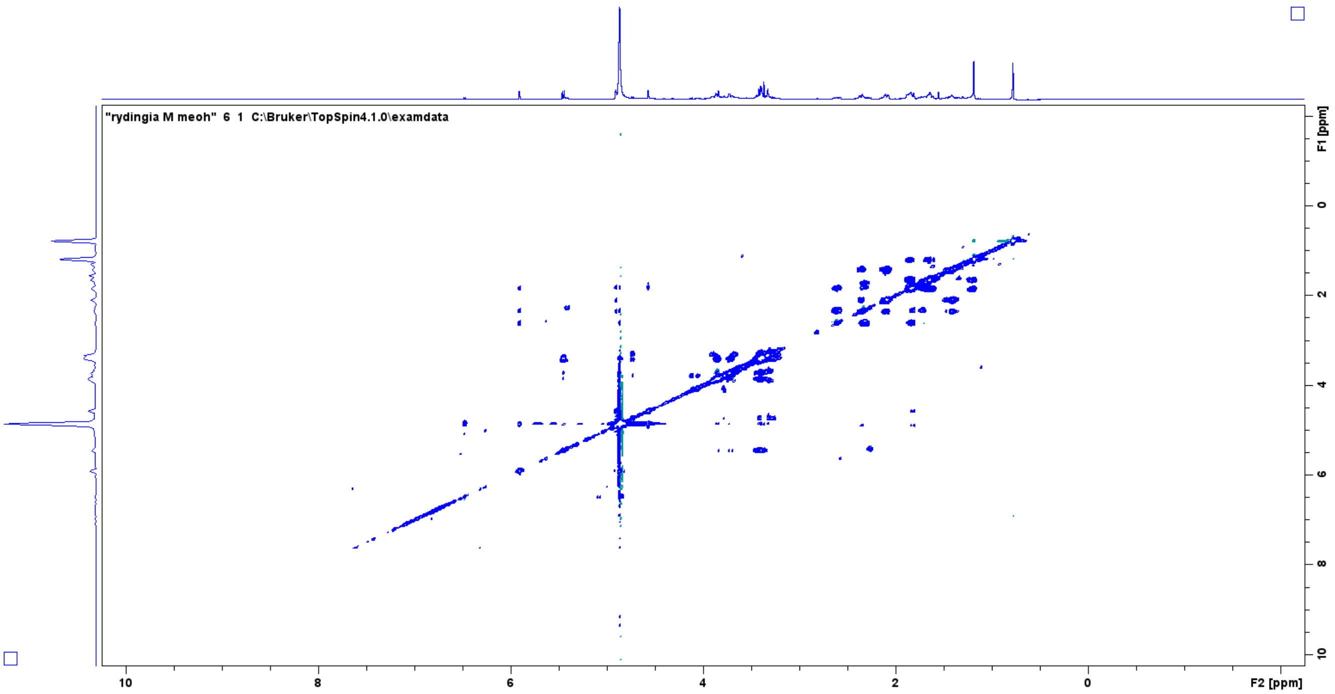

Figure S12: TOCSY of ent-labda-8(17),13-dien-18-oic acid-15,16-glucopyranoside

Table S3: NMR assignment for antirrhinoside in deuterated methanol

Position	δH	δC
1	5.41 d, *J*=8.5	94.6
3	6.48, d, *J*=6.1	142.3
4	4.84, d, under methanol signal	102.9
5	3.86 m	78.4
6	3.85 m	77.1
7	3.34 m	64.0
8	-	65.7
9	2.27 d, *J*=8.5	51.5
10	1.56 s	16.6
1′	5.43 d, *J*=8.2	94.0
2′	3.37	72.8
3′	3.39	78.9
4′	3.30	71.0
5′	3.38	77.1
6′	3.90-3.73	61.4

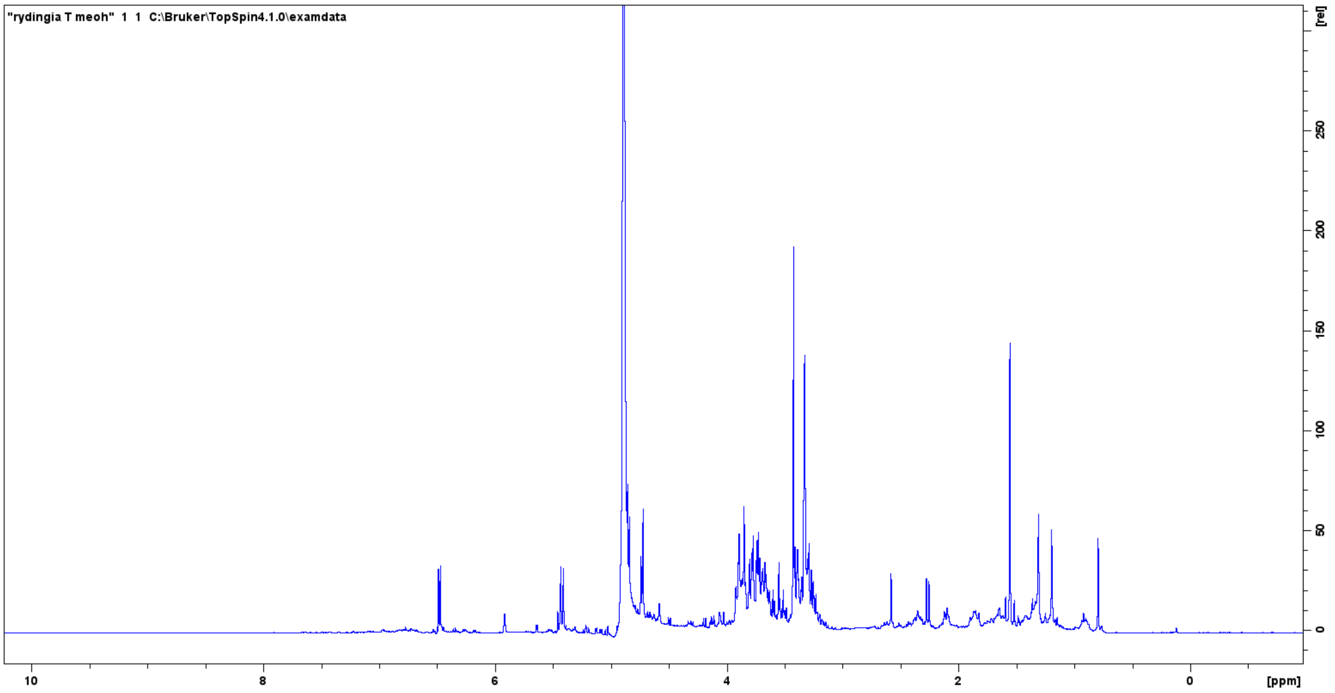

Figure S13: ^{1}H NMR of antirrhinoside

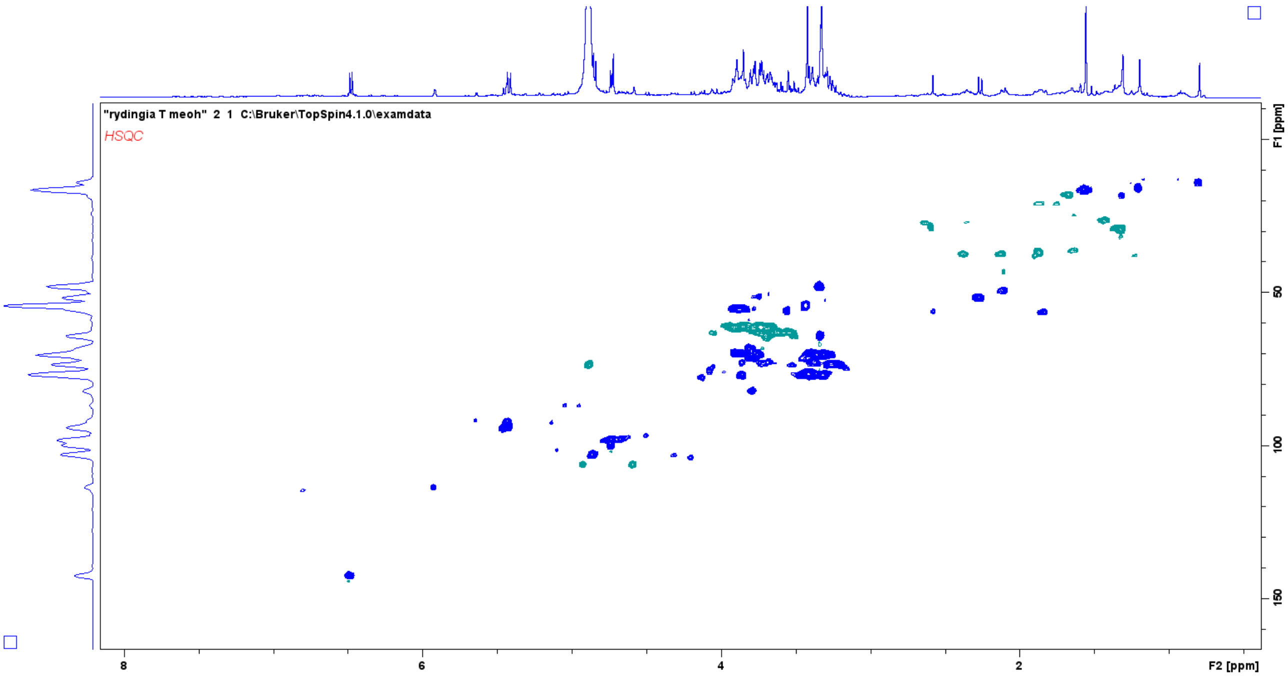

Figure S14: HSQC of antirrhinoside

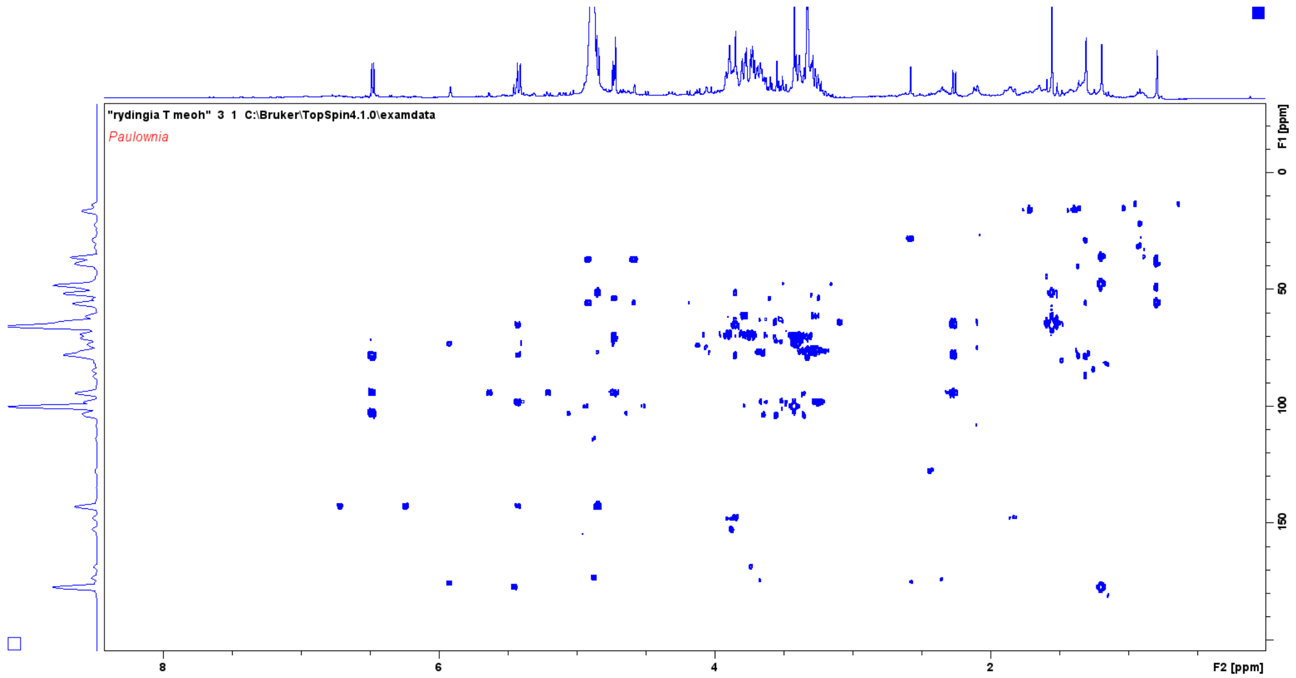

Figure S15: HMBC of antirrhinoside

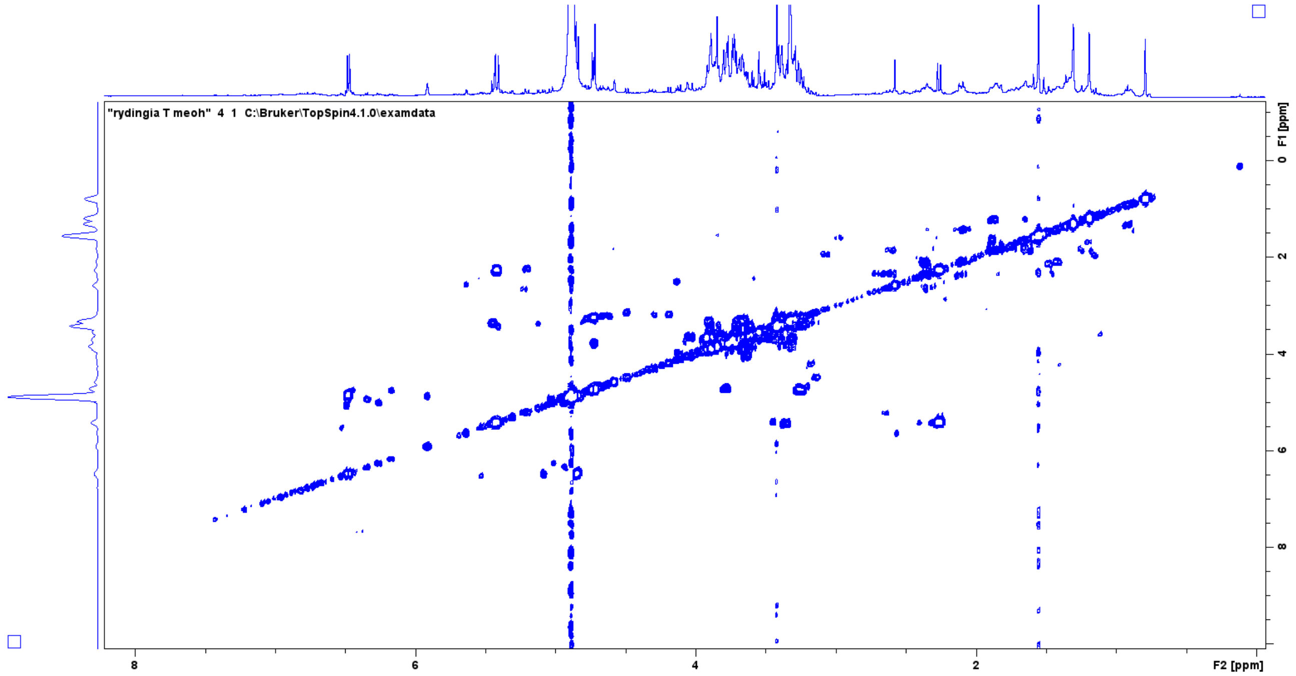

Figure S16: COSY of antirrhinoside

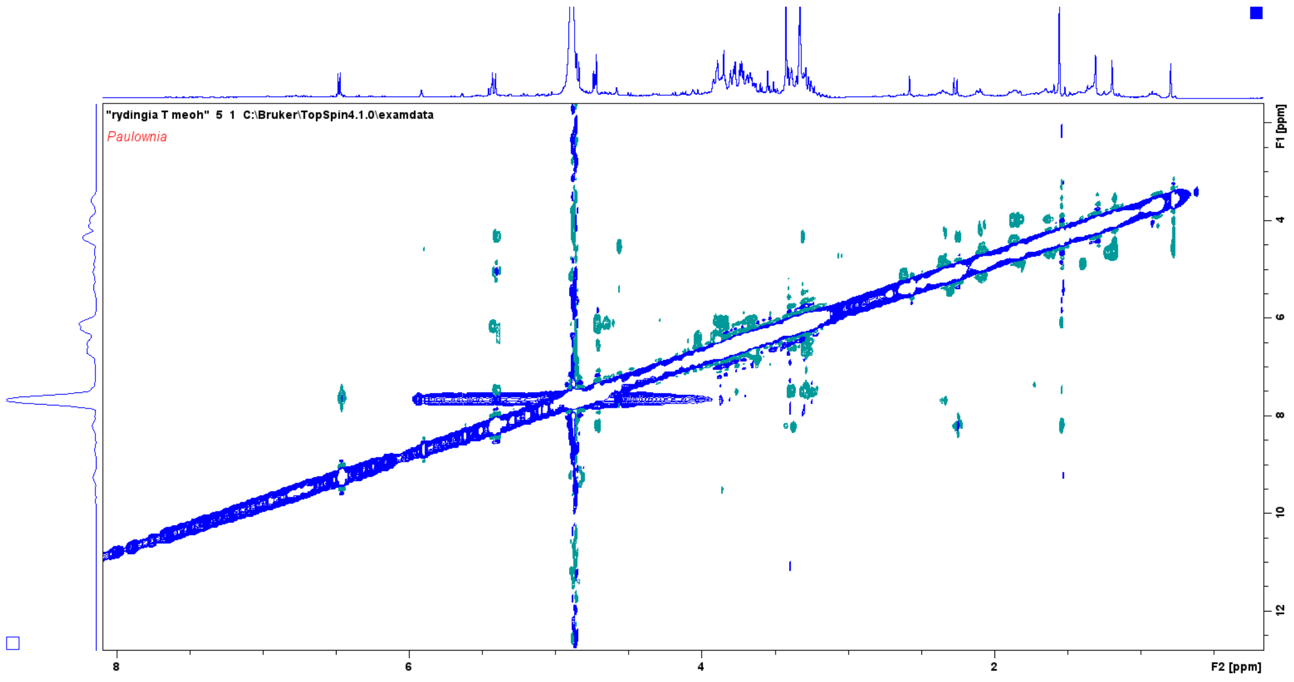

Figure S17: NOESY of antirrhinoside

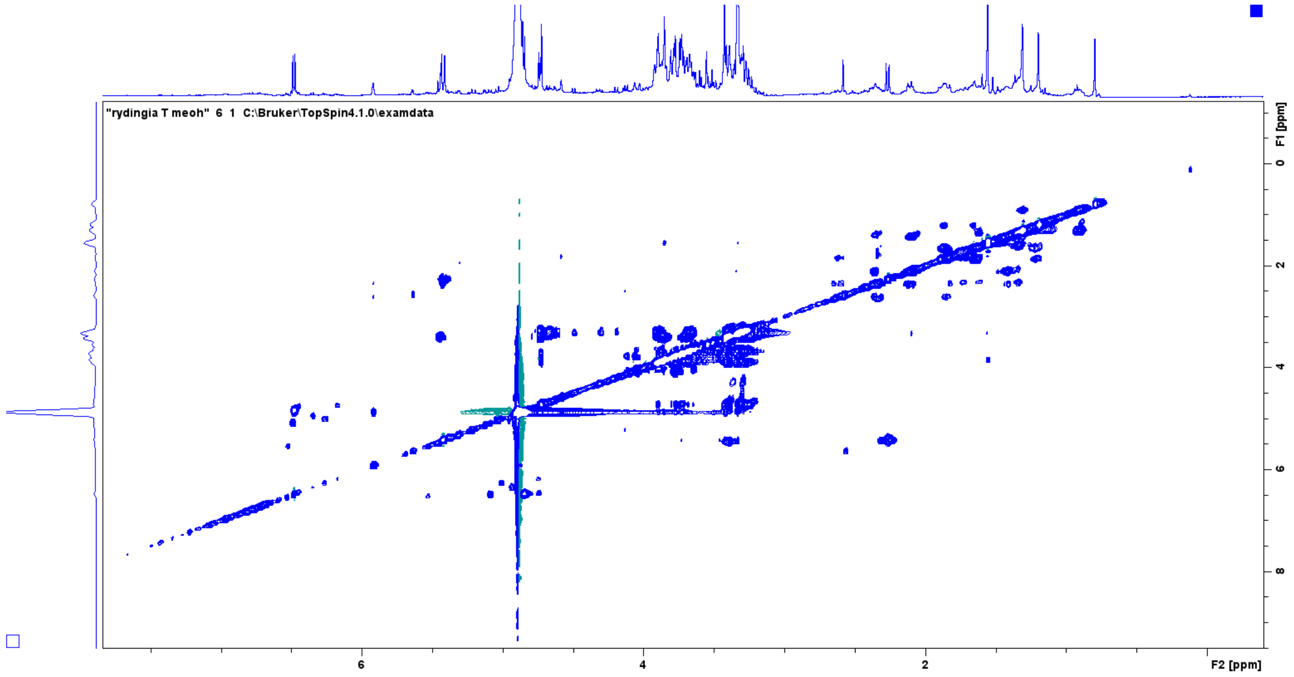

Figure S18: TOCSY of antirrhinoside
